# Risk scores for type 2 diabetes mellitus in Latin America: a systematic review of population‐based studies

**DOI:** 10.1111/dme.14114

**Published:** 2019-09-06

**Authors:** R. M. Carrillo‐Larco, D. J. Aparcana‐Granda, J. R. Mejia, N. C. Barengo, A. Bernabe‐Ortiz

**Affiliations:** ^1^ Department of Epidemiology and Biostatistics School of Public Health Imperial College London London UK; ^2^ CRONICAS Centre of Excellence in Chronic Diseases Universidad Peruana Cayetano Heredia Lima Perú; ^3^ Universidad Científica del Sur Lima Perú; ^4^ Centro de Estudios de Poblacion Universidad Catolica los Ángeles de Chimbote (ULADECHCatolica) Chimbote Perú; ^5^ Facultad de Medicina Humana Universidad Nacional del Centro del Perú Huancayo Perú; ^6^ Department of Medical and Population Health Sciences Research Herbert Wertheim College of Medicine Florida International University Miami FL USA; ^7^ Department of Public Health Faculty of Medicine University of Helsinki Helsinki Finland; ^8^ Faculty of Medicine Riga Stradins University Riga Latvia

## Abstract

**Aim:**

To summarize the evidence on diabetes risk scores for Latin American populations.

**Methods:**

A systematic review was conducted (CRD42019122306) looking for diagnostic and prognostic models for type 2 diabetes mellitus among randomly selected adults in Latin America. Five databases (LILACS, Scopus, MEDLINE, Embase and Global Health) were searched. type 2 diabetes mellitus was defined using at least one blood biomarker and the reports needed to include information on the development and/or validation of a multivariable regression model. Risk of bias was assessed using the PROBAST guidelines.

**Results:**

Of the 1500 reports identified, 11 were studied in detail and five were included in the qualitative analysis. Two reports were from Mexico, two from Peru and one from Brazil. The number of diabetes cases varied from 48 to 207 in the derivations models, and between 29 and 582 in the validation models. The most common predictors were age, waist circumference and family history of diabetes, and only one study used oral glucose tolerance test as the outcome. The discrimination performance across studies was ~ 70% (range: 66–72%) as per the area under the receiving‐operator curve, the highest metric was always the negative predictive value. Sensitivity was always higher than specificity.

**Conclusion:**

There is no evidence to support the use of one risk score throughout Latin America. The development, validation and implementation of risk scores should be a research and public health priority in Latin America to improve type 2 diabetes mellitus screening and prevention.

## What's new?

- Risk scores are tools that could support screening, diagnosis and prognosis decisions in clinical medicine and public health.
- Risk scores for undiagnosed diabetes or to predict diabetes are available worldwide with a few in Latin America. However, the characteristics of risk scores available for Latin America, their performance, pitfalls and other attributes have not been summarized or appraised.
- A lack of synthesized information makes it difficult to understand the strengths and limitations of the available tools, hampering their implementation in clinical and screening guidelines.
- We conducted a thorough search for risk scores for type 2 diabetes developed in Latin America, providing the clinical and public health communities with evidence to inform their decisions regarding these risk scores.
- Local and regional health organizations could recommend one risk score or foster the development of a stronger tool to overcome the limitations signalled herein.

**What's new?**

- Risk scores are tools that could support screening, diagnosis and prognosis decisions in clinical medicine and public health.
- Risk scores for undiagnosed diabetes or to predict diabetes are available worldwide with a few in Latin America. However, the characteristics of risk scores available for Latin America, their performance, pitfalls and other attributes have not been summarized or appraised.
- A lack of synthesized information makes it difficult to understand the strengths and limitations of the available tools, hampering their implementation in clinical and screening guidelines.
- We conducted a thorough search for risk scores for type 2 diabetes developed in Latin America, providing the clinical and public health communities with evidence to inform their decisions regarding these risk scores.
- Local and regional health organizations could recommend one risk score or foster the development of a stronger tool to overcome the limitations signalled herein.

## Introduction

Type 2 diabetes mellitus is a leading cause of morbidity, disability and mortality worldwide 1, 2, 3, disproportionally affecting low‐ and middle‐income countries in Latin America 4. In addition, type 2 diabetes mellitus imposes a heavy financial burden on local healthcare systems 5. Therefore, the increasing number of newly detected type 2 diabetes mellitus cases creates challenges for low‐ and middle‐income countries 6. The United Nations/World Health Organization have set several goals to reduce the burden of non‐communicable diseases, including a 0% increase in diabetes 7. For Latin America, in particular, the Pan American Health Organization has issued policies and guidelines for the control and prevention of diabetes 8. Epidemiological evidence along with the active participation of international health organizations, support the relevance of identifying pragmatic strategies to reduce type 2 diabetes mellitus burden at the population level.

A pragmatic, although still challenging solution is the early identification of people with type 2 diabetes or those at high risk of developing type 2 diabetes so that non‐pharmacological and pharmacological prevention strategies can be initiated. Diagnostic and prognostic models such as risk scores are convenient for this purpose and yet their use is limited to the population for which they were developed, hence internal and external validation before application in new populations are recommended. Although there have been previous efforts to synthesize available risk scores globally 9, 10, 11, 12, even focusing on Latin American populations in the USA 13, scientific information on type 2 diabetes risk scores in Latin American countries has been limited. Therefore, whether there are scientifically validated type 2 diabetes mellitus risk scores for populations in Latin America remains unknown.

We aimed to critically review the current scientific evidence on developed diabetes risk scores for Latin American populations. In so doing, we provide a list of risk scores that could be further studied in different Latin American countries, used by practitioners in countries where the models were developed, or integrated by guideline/policy‐makers in the current standard of practice for diabetes screening at the population level. Emphasis is placed on tools developed for the general population because of their ability to be used in different communities, thus benefiting populations beyond those accessing the health system 14, 15, 16.

## Methods

### Protocol and registration

This review is a systematic and critical appraisal of the scientific literature following PRISMA guidelines and registered at PROSPERO (CRD42019122306) 17. The review framework adheres to international recommendations for systematic reviews of prediction models and followed the CHARMS strategy 18, 19.

### Eligibility criteria

Eligibility criteria for studies following the CHARMS checklist are given in Table 1. In brief, we searched both diagnostic and prognostic models aiming to inform general practitioners (GPs), clinicians, researchers and the general population about their current type 2 diabetes status (i.e. diagnostic) or future risk (i.e. prognostic). The studies could present results for models with or without external validation. The target population was adults in Latin America with no restrictions on age.

**Table 1:** Criteria to guide the literature search and selection criteria

Concept	Criteria
Prognostic or diagnostic?	Both, this review focuses on diagnostic and prognostic risk scores for type 2 diabetes mellitus
Scope	Diagnostic/prognostic models to inform physicians, researchers and general population about their current type 2 diabetes mellitus status (i.e. diagnostic) or risk of type 2 diabetes mellitus in the future (i.e. prognostic)
Type of prediction modelling studies	Focus on the three types: (i) diagnostic/prognostic models with external validation, (ii) diagnostic/prognostic models without external validation, and (iii) diagnostic/prognostic models validation
Target population to whom the prediction model applies	General adult population in Latin America and the Caribbean; no age or gender restrictions
Outcome to be predicted	type 2 diabetes mellitus (diagnostic or prognostic)
Time span of prediction	Any; prognostic models will not be included/excluded based on the prediction time span
Intended moment of using the model	Diagnostic/prediction models to be used in asymptomatic adults in Latin America to ascertain current type 2 diabetes mellitus status (i.e. diagnostic) or future risk of type 2 diabetes mellitus (i.e. prognostic); these models could be used for research purposes, screening and treatment allocation in primary prevention

Based on the CHARMS checklist. 19

### Information sources

Five search engines were used systematically: LILACS, Scopus, MEDLINE, Embase and Global Health; the last three through Ovid. The search was conducted on 15 January 2019 with no time or language restrictions. The search terms used are given in Appendices S1–S3.

### Study selection

Reports were selected if the study population included men and women who were both from and living in any Latin American country. Thus, studies including Latin American populations outside Latin America or those including only foreigners living in Latin America were excluded. To be included in this review, the study participants had to be a randomly selected sample of the general population. Studies of convenience samples were excluded. Furthermore, studies including a specific subsample of people (e.g. studies in obese or hypertensive people) as well as hospital‐based samples were excluded. The outcome of interest was previously undiagnosed type 2 diabetes mellitus, defined using at least one biomarker such as fasting glucose, random glucose, oral glucose tolerance test or HbA_1c_. Studies in which the outcome was defined solely based on self‐reported diagnosis were excluded. Reports needed to present the development and/or validation procedures of a multivariable model. Thus, studies assessing the diagnostic or predictive power of one variable or biomarker alone were excluded. In addition, both cross‐sectional and cohort studies were included.

### Data collection process

Results from the literature search were downloaded into EndNote and duplicates were removed. All unique results were uploaded to Rayyan 20, an online systematic review tool, whereby titles and abstracts were independently screened by two reviewers (pairwise combinations between RMC‐L, DJA‐G, JRM) and disagreements were solved by consensus or by a third party (AB‐O). Before screening, all reviewers underwent a standardization process. Reports selected from the screening phase were studied in detail by two reviewers independently (RMC‐L, DJA‐G, JRM), and disagreements were solved by consensus or by a third party (AB‐O). These processes led to the selection of reports for inclusion in the qualitative summary, from which key information was extracted onto a data collection form developed by the authors based on international guidelines for systematic reviews of prognosis models (CHARMS checklist) 18, 19. The data collection form was not modified afterwards. Information was extracted by one reviewer (RMC‐L) and independently verified by another (AB‐O); disagreements were solved by consensus.

### Risk of bias of individual studies

Risk of bias was assessed following the PROBAST recommendations, a risk of bias assessment tool developed exclusively for prognosis models 21, 22. This process was conducted by two reviewers independently (DJA‐G and JRM) and verified by a third (AB‐O).

### Synthesis of results

Only a qualitative synthesis was conducted. A quantitative synthesis was not possible because of the small number of reports using the same variables in the prediction models.

This study was classified as low risk because no human participants were studied. This is a systematic review of the scientific literature, which is public and can be accessed freely.

## Results

**Figure 1:**
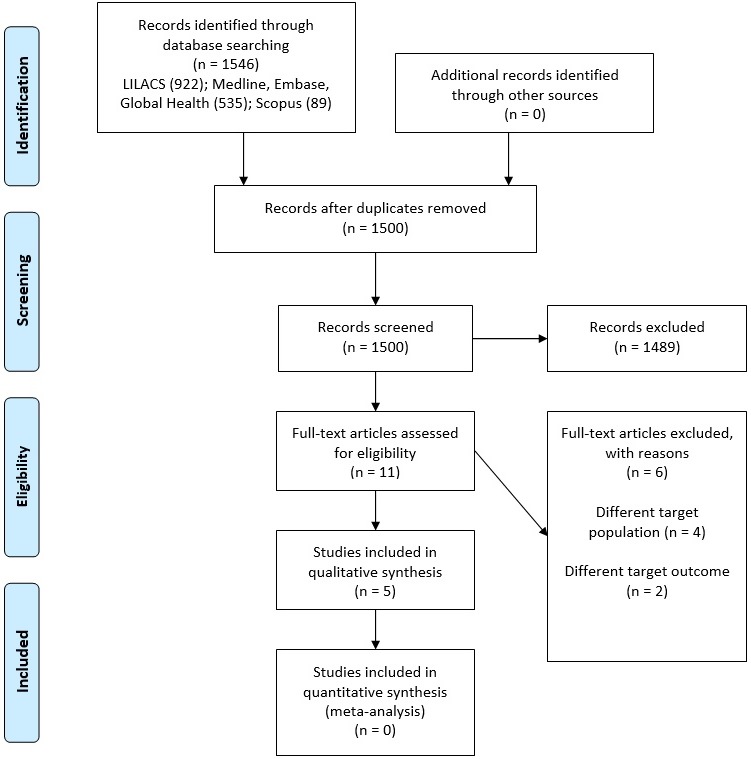
Flowchart of the study selection process.

The initial search yielded 1546 results; 1500 titles and abstracts were screened and 11 reports were studied in full. Five reports were included in the qualitative synthesis (Fig. 1) 23, 24, 25, 26, 27.

### What has been done?

In 2018, Bernabe‐Ortiz and colleagues derived a simplified version of the FINDRISC, and validated the original FINDRISC, the Latin America‐FINDRISC and the Peruvian Risk Score, i.e. they provided estimates for four models (one derivation and three validations) 23. In 2016, Bernabe‐Ortiz *et al*. derived a diagnostic model, which was validated externally, using both cross‐sectional and prospective data, i.e. they provided estimates for three models (one derivation and two validations) 24. In 2018, Félix‐Martinez and Godínez‐Fernández derived and validated two models using cross‐sectional data collected in 2006 and 2012, i.e. they provided estimates for four models (two derivations and two validations) 25. In 2010, Guerrero‐Romero and Rodríguez‐Morán derived a model using cross‐sectional data and validated it in prospective data, i.e. provided estimates for two models (one derivation and one validation) 26. Finally, in 2009, Pires de Sousa and colleagues derived and validated a diagnostic model, i.e. they provided estimates for two models (one derivation and one validation) 27. Overall, six models were derived and nine underwent validation analysis.

### General characteristics

Two of the five reports studied people in Peru 23, 24, two studied Mexicans 25, 26, and one was conducted in Brazil 27. The oldest analysed data was collected in 1996 26, and the remainder of the studies used data collected after 2000 23, 24, 25, 27. The mean age of the participants in the derivation models ranged from 42 to 50 years, and the proportion of men varied from 38% to 51% 23, 24, 25, 26, 27. The mean age of people analysed in the validation models ranged from 40 to 55 years, and the proportion of men ranged from 25% to 49% (Appendix S4) 23, 24, 25, 26, 27.

The sample size analysed to derive the diagnostic models ranged from 711 26 to 6995 people 25, and from 438 26 to 28 913 25 for the validation models. The number of diabetes cases varied greatly in the derivation models, from 48 24 to 207 25, with only two derivations models having ≥ 100 events; the equivalent numbers in the validation models were 29 27 and 582 25, with two derivation models having ≥ 100 events. Of note, this information (number of outcome events or diabetes cases) could not be extracted from Guerrero‐Romero and Rodríguez‐Morán 26. The ratio of outcome events per number of candidate predictors in the derivation analyses ranged from 3.43 24 to 15.92 25. Across all reports, missing data were handled by conducting a complete‐case analysis 23, 24, 25, 27, although this information was not available in Guerrero‐Romero and Rodríguez‐Morán's study 26 (Appendix S4)

**Figure 2:**
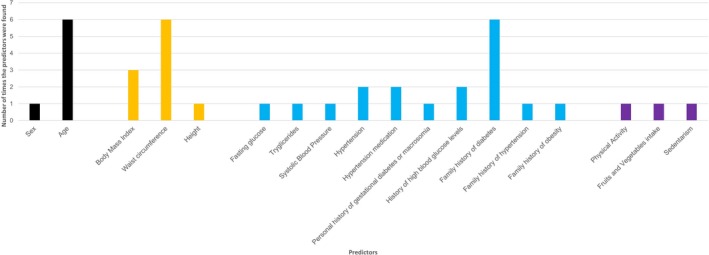
Predictors included in the final diagnosis and prognosis models. The colours of the bars identify the underlying characteristic of predictors inherent to: the subject (black), anthropometrics (orange), clinical assessment and history (blue) and lifestyle‐related behaviours (purple).

All derivations models used a logistic regression analysis 23, 24, 25, 26, 27. In all but one report 26, it was clear that preselection of predictors was conducted (i.e. choosing the final list of predictors based on statistical significance), mostly following a stepwise backward selection approach (Appendix S4) 23, 24, 25, 27. In Félix‐Martinez and Godínez‐Fernández's work, numerical variables were not categorized 25, but this approach was followed in the other studies 23, 24, 26, 27. As shown in Fig. 2, the most common predictors used in the models were: age, waist circumference and family history of diabetes (Appendix S4) 23, 24, 25, 26, 27.

### Outcome ascertainment

Across reports, the outcome was new type 2 diabetes cases based on standard laboratory procedures. It was not possible to extract the definition used to diagnose new diabetes cases from Guerrero‐Romero and Rodríguez‐Morán's work 26. The other reports relied mostly on fasting plasma glucose ≥ 7.0 mmol/l (126 mg/dl) 23, 24, 25, 27. In addition to fasting plasma glucose, Bernabe‐Ortiz *et al*. also used 2‐h plasma glucose ≥ 11.1 mmol/l (≥ 200 mg/dl) 23. Félix‐Martinez and Godínez‐Fernández 25 also defined new diabetes cases according to random glucose ≥ 11.1 mmol/l (≥ 200 mg/dl) (Appendix S4).

### Model performance

**Figure 3:**
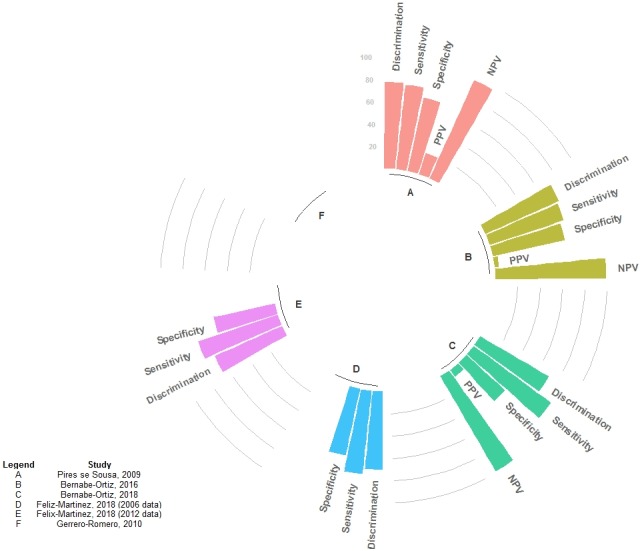
Performance metrics of the derivation models. Guerrero‐Romero and Rodríguez‐Morán 26 did not provide these details for the derivation models, thus it was left blank. Pires de Sousa *et al*. 27 presented these metrics for several thresholds (Appendix S4), the ones shown here correspond to the cut‐off point with sensitivity closest to the pre‐specified value. Bernabe‐Ortiz *et al*. 24 presented these metrics for several thresholds (Appendix S4), the ones shown here correspond to those with the best Youden Index. A, Pires de Souza, 2009; B, Bernabe‐Ortiz, 2016; C, Bernabe‐Ortiz, 2018; D, Félix‐Martinez, 2018 (2006 data); E, Félix‐Martinez, 2018 (2012 data); F, Guerrero‐Romero, 2010. NPV, negative predictive value; PPV, positive predictive value.

Figure 3 shows the performance metrics for each derivation model as presented in the original reports (Appendix S4) 23, 24, 25, 26, 27. Discrimination performance across studies was ~ 70% as per the area under the receiving‐operator curve, ranging from 66% 25 to 72% 24, 27. Where reported, the negative predictive value was the best metric, achieving almost 100% 23, 24, 27. Sensitivity was always larger than specificity 23, 24, 25, 27 and the largest absolute difference was 39.2% (sensitivity, 85.9%; specificity, 46.7%) 23.

**Figure 4:**
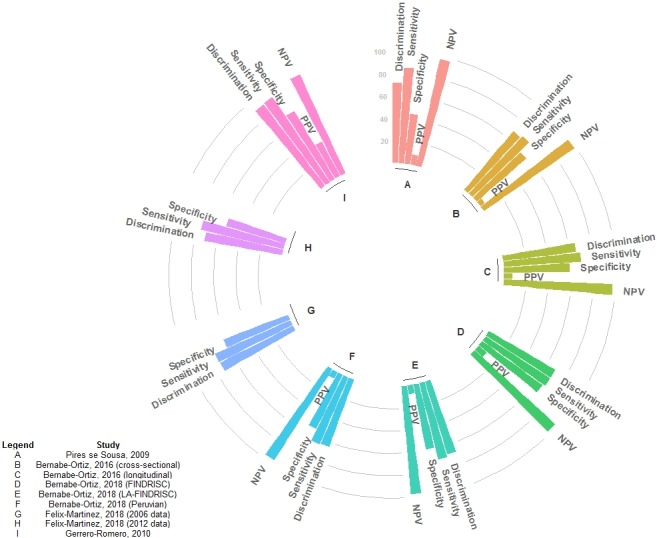
Performance metrics of the validation models. Pires de Sousa *et al*. 27 presented these metrics for several thresholds (Appendix S4), the ones shown here correspond to the cut‐off point with the sensitivity closest to the pre‐specified value. Bernabe‐Ortiz *et al*. 24 presented these metrics for several thresholds (Appendix S4), the ones shown here correspond to those with the best Youden Index. A, Pires de Souza, 2009; B, Bernabe‐Ortiz, 2016 (cross‐sectional); C, Bernabe‐Ortiz, 2016 (longitudinal); D, Bernabe‐Ortiz, 2018 (FINDRISC); E, Bernabe‐Ortiz, 2018 (LA‐FINDRISC); F Bernabe‐Ortiz, 2018 (Peruvian); G, Félix‐Martinez, 2018 (2006 data); H, Félix‐Martinez, 2018 (2012 data); I, Guerrero‐Romero, 2010. NPV, negative predictive value; PPV, positive predictive value.

Figure 4 shows the performance metrics for the validation models as reported originally (Appendix S4) 23, 24, 25, 26, 27. Discrimination performance ranged from 64.0% 25 to 91.0% 26. The best performance metric was the negative predictive value, and sensitivity was always larger than specificity (Appendix S4) 23, 24, 25, 26, 27. In both prediction and validation analyses, calibration metrics or plots were not presented, though Bernabe‐Ortiz *et al*. reported the Hosmer–Lemeshow *P*‐value (0.21) 24.

### Risk of bias

Table 2 summarizes the risk of bias ascertainment, and full details are provided in Appendix S5. Across all reports, the participants’ criterion in the risk of bias assessment revealed a low risk of bias; by contrast, the analysis criterion showed high risk of bias mostly because of few numbers of outcome events (i.e. diabetes cases) and conducting complete‐cases analysis instead of performing imputation methods. Across summarized studies, there was a low concern regarding applicability as models were created from population‐based studies.

**Table 2:** Risk of bias assessment of individual diagnostic/prognostic models (PROBAST)

Study; first author, date	Objective	Risk of bias				Applicability			Overall	
		Participants	Predictors	Outcome	Analysis	Participants	Predictors	Outcome	Risk of bias	applicability
Pires de Sousa, 2009 27	Derivation	+	+	+	−	+	+	+	?	+
Pires de Sousa, 2009 27	Validation	+	+	+	−	+	+	+	?	+
Bernabe‐Ortiz, 2016 27	Derivation	+	+	+	−	+	+	+	?	+
Bernabe‐Ortiz, 2016 27	Validation	+	+	+	−	+	+	+	?	+
Bernabe‐Ortiz, 2018 (FINDRISC) 23	Validation	+	+	+	−	+	+	+	−	+
Bernabe‐Ortiz, 2018 (Latin America‐FINDRISC) 23	Validation	+	+	+	−	+	+	+	−	+
Bernabe‐Ortiz, 2018 (Peruvian) 23	Validation	+	+	+	−	+	+	+	−	+
Bernabe‐Ortiz, 2018 (Simplified FINDRISC) 23	Derivation	+	+	+	−	+	+	+	−	+
Félix‐Martinez, 2018 (NHNS‐2006) 25	Derivation	+	?	?	−	+	+	+	−	+
Félix‐Martinez, 2018 (NHNS‐2012) 25	Derivation	+	?	?	−	+	+	+	−	+
Félix‐Martinez, 2018 (NHNS‐2006) 25	Validation	+	?	?	−	+	+	+	−	+
Félix‐Martinez, 2018 (NHNS‐2012) 25	Validation	+	?	?	−	+	+	+	−	+
Guerrero‐Romero, 2010 26	Derivation	?	−	−	−	?	‐	−	−	−
Guerrero‐Romero, 2010 26	Validation	+	−	−	−	+	−	−	−	−

PROBAST, Prediction model Risk of Bias ASsessment Tool 21, 22; +, low risk of bias/low concern regarding applicability; −, high risk of bias/high concern regarding applicability; ?, unclear risk of bias/unclear concern regarding applicability.

## Discussion

### Main findings

This systematic review of the literature synthesized the available risk scores for type 2 diabetes mellitus that can be used in Latin American general populations and countries, providing evidence and tools for practitioners as well as guideline/policy‐makers across Latin America. Five reports from three countries (Brazil, Mexico and Peru) were summarized 23, 24, 25, 26, 27, which developed five diagnostic tools; two of them also conducted a longitudinal assessment 24, 26. In addition, these five reports provided results for the validation of nine models 23, 24, 25, 26, 27. Although discrimination estimates were largely acceptable, calibration metrics were not reported. The negative predictive value was the highest metric across risk scores 23, 24, 25, 26, 27. Even though several type 2 diabetes mellitus risk scores have been developed for Latin American populations, few have followed optimal analytical approaches regarding internal and external validation. For countries (Brazil, Mexico and Peru) where risk scores were generated and validated both cross‐sectionally and prospectively, there is enough scientific evidence to implement them as part of the standard of care for type 2 diabetes mellitus screening at the population level.

### Limitations of the review

This is a sound methodological review following international guidelines for the systematic reviews of prognosis models 18, 19, 21, 22. In addition, we used several search engines including ones based in Latin America, hence most, if not all, available evidence should have been retrieved. However, we did not systematically search grey literature, e.g. dissertations. We argue that this potential limitation would not change our overall findings and conclusions, because these sources would usually not retrieve population‐based studies and would have the same or more methodological issues.

### Limitations of the selected reports

Most of the reports ascertained the outcome based on fasting glucose, yet one effort in Peru also used oral glucose tolerance test 23.

It could be argued that results based on fasting glucose, or any single biomarker, could lead to underestimation, i.e. some cases might have not been detected. Nonetheless, we need to acknowledge that these studies were conducted in low‐ and middle‐income countries, sometimes in rural areas, were laboratory facilities to analyse a wider range of biomarkers is limited. In any case, this limitation does not invalidate the results, but rather invites additional investigations to further confirm them using more/other biomarkers. Furthermore, most of the selected reports followed a cross‐sectional design, which is not suitable for assessing prognostic models (i.e. long‐term outcomes). In addition, the study populations were rather young, which further limits the implementation of the available tools in very young individuals (e.g. adolescent or early adulthood) as well as among the elderly.

There were three main methodological limitations: (i) continuous predictors were categorized, (ii) there was preselection of the predictors, and (iii) some studies included a limited number of diabetes cases. The first two limitations have been identified as common but suboptimal approaches that hamper the prediction accuracy of the models 28. Some authors may argue that categorizing continuous predictors helps to make the risk score friendlier thus fostering their use. Whether this argument supersedes the statistical limitations remains unknown. Nevertheless, there are other ways to make the risk scores more accessible such as the use of mobile apps that could include a ‘complex’ algorithm without compromising statistical power. Alternatively, a spreadsheet could accompany the main report as supplementary material, also containing a ‘complex’ algorithm ready to be used. Preselection of predictors was a common practice, following a backward elimination technique 23, 24, 25, 27. This could lead to the omission of important predictors that by chance, are not statistically associated with the outcome in the training data set; moreover, this could lead to over‐fitting the risk model 29. A general recommendation could be to conduct a systematic review of available models in the field to identify the most common and relevant predictors; alternatively, expert knowledge should be included rather than statistical significance alone. Our work could help to overcome this limitation for future studies. We have summarized the most common predictors, so that future efforts could select these instead of ‘sampling’ within a pool of variables available in the data. The number of predictors was small in some derivation models; most importantly, this was also the case in the validation models. It has been suggested that for external validation, at least 100 events should be available 30. An additional methodological limitation, although one that has little impact in the selected studies, was analysing a complete‐case data set, i.e. not conducting methods to account for the missing observations. Multiple imputation techniques still seem to be conducted poorly or not be particularly popular among Latin American health data analysis.

Several metrics for the performance of the risk scores were reported, although calibration estimates were not available. Calibration is important because it tells us whether the prediction computed by the model agrees with what is actually observed; in other words, a poor calibration could result in overestimation (when the model predicts higher risk than the actual observed risk) or underestimation (when the model predicts lower risk than the actual observed risk) 29, 31. Although Bernabe‐Ortiz and colleagues reported the Hosmer–Lemeshow *P*‐value 24, further details such as a calibration plot comparing observed vs. predicted cases were missing. The absence of this performance metric but the presentation of other clinically relevant metrics such as sensitivity, specificity and negative/positive predictive values, highlight a need for further training in diagnostic/prediction models analysis. Regarding negative/predictive values, it is relevant to signal that these depend on the underlying prevalence in the population; therefore, these metrics should be interpreted in line with the prevalence estimates and would not be useful to compare prediction models across countries with very different prevalence rates. Given the relevance that risk scores may have in clinical medicine and public health, strengthening the analytical skills in this field appears to be necessary. Even though friendly technical literature is available 29, 32, 33, 34, 35, 36, the equivalent in Spanish, the language mostly spoken throughout Latin America, is limited.

Because the Transparent Reporting of a multivariable prediction model for Individual Prognosis or Diagnosis (TRIPOD) statement was published in 2015 37, 38, studies published before that date could not have adhered to this reporting checklist. Studies published after, by contrast, could have adhered to TRIPOD but probably did not because they were unaware of it, suggesting a lack of experience in the field and poor penetration of this statement across professions and regions. Another limitation regarding presentation of the results was the fact that only one study reported the baseline risk, i.e. the intercept of the logistic regression 25. This parameter is not generally reported and can make it difficult for other researchers to recalibrate these tools for other populations or countries.

Overall, the synthesized prediction models exhibited some methodological limitations. Although these do not invalidate the results, they further support the need to improve this research area in Latin America, for diabetes and other conditions, including several non‐communicable diseases. Conducting sound and methodologically robust analyses is key to taking advantage of all the available data and produce better tools that could be easily scaled to clinical medicine and supported by guidelines or policies.

### Additional evidence

This review focused on population‐based studies with random sampling, although this does not mean that studies following different sampling methods are of little relevance. To develop risk scores, random samples of the general population are not essential. Reports with different sampling criteria have provided valuable information and pragmatic tools for Latin American countries, and thus deserve to be acknowledged as well.

In Mexico, Rojas‐Martinez *et al*. using data of a cohort of public and private servants developed a risk score for undiagnosed diabetes; although the external validation was conducted on a population‐based sample, this endeavour was not selected for the main synthesis because the model was generated in a closed population 39. Their score yielded a discrimination of 60% in men and 63% in women, specificity was larger than sensitivity, and the negative predictive value was the largest metric 39. In addition, the authors compared this new tool with the one currently recommended in Mexico, concluding that the new one performed better 39. This work signalled that additional research on prediction models benefiting of new and larger data could be useful to improve and update current guidelines.

In Colombia, Barengo and colleagues analysed data of an insurance company to develop a risk score for undiagnosed type 2 diabetes mellitus 40. Their model had a discrimination of 74%, slightly higher than the internationally known FINDRISC (73%) 40. However, this study was not tested externally 40, leaving room for further validation in the general population. Other Colombian researchers have also tested the accuracy of the FINDRISC score, this time using data for people at a primary care facility 41. They reported a discrimination of 72% in women and 75% in men for undiagnosed type 2 diabetes mellitus 41; these numbers for incident diabetes were 68% in women and 72% in men 41. Although these two Colombian experiences should undergo further validation, they signal that available risk scores have a relevant prediction accuracy that could provide valuable tools to improve the early diagnosis of type 2 diabetes mellitus in Colombia.

A recent study in Venezuela also tested the Latin American version of the FINDRISC score, concluding that people above the proposed threshold must have an additional diagnostic test, e.g. oral glucose tolerance test 42. Because Munoz‐Gonzalez *et al*. studied volunteers attending cardiometabolic screening campaigns, further validation is warranted with a larger and more heterogenous study population.

Central America has contributed poorly to this systematic review, which calls researchers and health officers from this region to conduct studies to develop efficient approaches to early identify people with type 2 diabetes mellitus. However, Milton and collaborators developed a prognostic model benefiting from data of a primary care clinic; their model yielded a discrimination of 89% 43. Despite the limitations of this work, it is worth acknowledging that the model was intended for rural populations in Honduras, who have been underrepresented to date in the selected and discussed type 2 diabetes mellitus risk scores.

### Clinical and public health relevance

Clinical guidelines provide recommendations for type 2 diabetes mellitus screening. The Latin American guidelines, issued by the Latin America Diabetes Association (Asociacion Latinoamericana de Diabetes), recommends screening with fasting glucose if a person has one of more risk factors (e.g. overweight, abdominal obesity, family history of type 2 diabetes mellitus) 44. In addition, if a person is ≥ 45 years old, they should be screened with a fasting glucose test at least once every 5 years, although this could be more often depending on the co‐existence of other risk factors 44. Of note, this guideline also recommends the use of a validated risk score such as the FINDRISC, which could guide the decision on whether or not someone should be screened using fasting glucose 44. However, about one third of people with undetected type 2 diabetes have normal fasting glucose levels but 2‐h postprandial glucose values of > 200 mg/dl. Our work provides evidence on additional type 2 diabetes mellitus risk scores locally developed and validated in Latin America, thereby this and other guidelines can update their recommendations with strong regional evidence to secure better and more reliable diabetes screening in Latin American populations.

American guidelines also propose screening individuals with risk factors, and suggest using the American Diabetes Association risk test to inform the decision on who should undergo further diagnostic tests (e.g. fasting glucose) 45. Similarly, the Canadian guidelines propose screening people based on risk factors, or using the Canadian Diabetes Risk Assessment Questionnaire (CANRISK) 46. Importantly, both, the American and Canadian guidelines, include locally developed and validated risk scores. Furthermore, the Canadian guidelines offer a brief but solid preface on the relevance of using validated risk scores 46. By contrast, the Latin American guidelines simply suggest use of a ‘validated risk score’. Apparently, this general recommendation was made in the absence of a comprehensive list of available type 2 diabetes mellitus risk scores for Latin American populations. Thus, our systematic review fills this knowledge gap providing scientific evidence to improve regional‐ and country‐based guidelines for the detection of type 2 diabetes mellitus.

It may seem bold to seek one risk score for Latin America as a region, but still worth trying because it could bring great benefits in population screening and disease prevention. With relevant methods, as shown in cardiovascular medicine 47, 48, along with support from stakeholders and professional bodies, one or a series of country‐specific risk scores could be acceptable and strongly recommended throughout Latin America.

### Conclusions

This systematic review of risk scores for the diagnosis and prognosis of type 2 diabetes mellitus could not find compelling evidence to strongly support the use of one single diabetes risk score throughout Latin America. Conversely, there was good evidence to support the use of validated risk scores in Peru and Mexico, whereas further studies need to be conducted with a multi‐country or regional scope. Because risk scores could provide additional options to identify type 2 diabetes mellitus cases early, hence decreasing the burden of this disease, the development, validation and implementation of accurate risk scores should be a research and public health priority in Latin America and other low‐ and middle‐income regions.

## Funding sources

Strategic Award, Wellcome Trust‐Imperial College Centre for Global Health Research (100693/Z/12/Z). Imperial College London Wellcome Trust Institutional Strategic Support Fund [Global Health Clinical Research Training Fellowship] (294834/Z/16/Z ISSF ICL). Rodrigo M Carrillo‐Larco is supported by a Wellcome Trust International Training Fellowship (214185/Z/18/Z). The funder had no role in the conception of this work, neither in the preparation or presentation of results. The authors are responsible for the results and opinions in this work.

## Competing interests

None declared.

## Supporting information


**Appendix S1.** Search terms used in MEDLINE, Embase and Global Health (through Ovid).
**Appendix S2.** Search terms used in Scopus.
**Appendix S3.** Search terms used in LILACS.
**Appendix S4.** Data extraction by domain.
**Appendix S5.** Risk of bias assessment (PROBAST).
