# Supplementary material for: Risk scores for type 2 diabetes mellitus in Latin America: a systematic review of population‐based studies
Source: Diabet Med. 2019 Sep 6;36(12):1573–84. doi: 10.1111/dme.14114 (PMC6900051; doi:10.1111/dme.14114)
Supplement: Supplementary file 1 — Appendix S1. Search terms used in MEDLINE, Embase and Global Health (through Ovid). Appendix S2. Search terms used in Scopus. Appendix S3. Search terms used in LILACS. Appendix S4. Data extraction by domain. Appendix S5. Risk of bias assessment (PROBAST). [file DME-36-1573-s001.docx]

**Risk scores for Type 2 diabetes mellitus in Latin America: a systematic review of population-based studies**

R. M. Carrillo-Larco, D. J. Aparcana-Granda, J. R. Mejia, N. C. Barengo and A. Bernabe-Ortiz

# **Appendix S1 Search terms used in MEDLINE, Embase and Global Health (through Ovid)**

| 01 | type 2 diabetes.mp. |
| --- | --- |
| 02 | T2D*.mp. |
| 03 | exp Diabetes Mellitus/ |
| 04 | exp Diabetes Mellitus, Type 2/ |
| 05 | pre-diabetes.mp. |
| 06 | pre-diabetic.mp. |
| 07 | prediabetic state.mp. |
| 08 | diabetes.mp. |
| 09 | ("type 2" or type two or type ii or type II).mp. |
| 10 | 08 and 09 |
| 11 | 01 or 02 or 03 or 04 or 07 or 10 |
|  |  |
| 12 | risk assessment.mp. |
| 13 | risk functions.mp. |
| 14 | Risk Assessment/mt |
| 15 | risk equation$.mp. |
| 16 | risk chart?.mp. |
| 17 | (risk adj3 tool$).mp. |
| 18 | risk assessment function?.mp. |
| 19 | risk assessor.mp. |
| 20 | risk appraisal$.mp. |
| 21 | risk calculation$.mp. |
| 22 | risk calculator$.mp. |
| 23 | risk factor$ calculator$.mp. |
| 24 | risk factor$ calculation$.mp. |
| 25 | risk engine$.mp. |
| 26 | risk equation$.mp. |
| 27 | risk table$.mp. |
| 28 | risk threshold$.mp. |
| 29 | risk disc?.mp. |
| 30 | risk disk?.mp. |
| 31 | risk scoring method?.mp. |
| 32 | scoring scheme?.mp. |
| 33 | risk scoring system?.mp. |
| 34 | risk scal$.mp. |
| 35 | risk prediction?.mp. |
| 36 | risk algorith$.mp. |
| 37 | prediction model$.mp. |
| 38 | predictive instrument?.mp. |
| 39 | project$ risk?.mp. |
| 40 | predictive model?.mp. |
| 41 | scoring method$.mp. |
| 42 | (prediction$ adj3 method$).mp. |
| 43 | exp Risk Assessment/ |
| 44 | (risk? adj1 assess$).mp. |
| 45 | screening.mp. |
| 46 | diagnostic test.mp. |
| 47 | 12 or … 46 |
|  |  |
| 48 | Finnish Diabetes Risk Score.mp. |
| 49 | FINDRISC.mp. |
| 50 | Latin-American FINDRISC.mp. |
| 51 | LA-FINDRISC.mp. |
| 52 | 48 or 49 or 50 or 51 |
| 53 | 47 or 52 |
|  |  |
| 54 | ("Antigua and Barbuda" or "Argentina" or "Bahamas" or "Barbados" or "Belize" or "Bolivia" or "Brazil" or "United States Virgin Islands" or "British Virgin Islands" or "Chile" or "Colombia" or "Costa Rica" or "Cuba" or "Dominica" or "Dominican Republic" or "Ecuador" or "El Salvador" or "Grenada" or "Guatemala" or "Guyana" or "Haiti" or "Honduras" or "Jamaica" or "Mexico" or "Nicaragua" or "Panama" or "Paraguay" or "Peru" or "Puerto Rico" or "Saint Kitts and Nevis" or "Saint Lucia" or "Saint Vincent and the Grenadines" or "Suriname" or "Trinidad and Tobago" or "West Indies" or "Uruguay" or "Venezuela" or "Latin America" or latin amer$ or "South America" or south amer$ or "Central America" or central amer$ or "Caribbean Region").mp. |
|  |  |
| 55 | 11 and 53 and 54 |
| 56 | exp animals/ not humans.sh. |
| 57 | 55 not 56 |
| 58 | Remove duplicates from 57 |

# **Appendix S2 Search terms used in Scopus**

(TITLE-ABS-KEY(diabetes mellitus) OR TITLE-ABS-KEY (Diabetes Mellitus, Type 2) OR TITLE-ABS-KEY(diabetes type 2) OR TITLE-ABS-KEY (type 2 diabetes) OR TITLE-ABS-KEY(pre-diabetes) OR TITLE-ABS-KEY(pre-diabetic) OR TITLE-ABS-KEY(prediabetic state)) AND (TITLE-ABS-KEY(Risk Assessment) OR TITLE-ABS-KEY(risk? adj1 assess*) OR TITLE-ABS-KEY(risk function) OR TITLE-ABS-KEY(Risk Assessment) OR TITLE-ABS-KEY(risk functions) OR TITLE-ABS-KEY(risk equation*) OR TITLE-ABS-KEY(risk chart?) OR TITLE-ABS-KEY(risk adj3 tool*) OR TITLE-ABS-KEY(risk assessment function?) OR TITLE-ABS-KEY(risk assessor) OR TITLE-ABS-KEY(risk appraisal*) OR TITLE-ABS-KEY(risk calculation*) OR TITLE-ABS-KEY(risk calculator*) OR TITLE-ABS-KEY(risk factor* calculator*) OR TITLE-ABS-KEY(risk factor* calculation*) OR TITLE-ABS-KEY(risk engine*) OR TITLE-ABS-KEY(risk equation*) OR TITLE-ABS-KEY(risk table*) OR TITLE-ABS-KEY(risk threshold*) OR TITLE-ABS-KEY(risk disc?) OR TITLE-ABS-KEY(risk disk?) OR TITLE-ABS-KEY(risk scoring method?) OR TITLE-ABS-KEY(scoring scheme?) OR TITLE-ABS-KEY(risk scoring system?) OR TITLE-ABS-KEY(risk prediction?) OR TITLE-ABS-KEY(risk algorith*) OR TITLE-ABS-KEY(prediction model*) OR TITLE-ABS-KEY(predictive instrument?) OR TITLE-ABS-KEY(project* risk?) OR TITLE-ABS-KEY(predictive model?) OR TITLE-ABS-KEY(scoring method*) OR TITLE-ABS-KEY(prediction* adj3 method*) OR TITLE-ABS-KEY(screening) OR TITLE-ABS-KEY(risk scal*) OR TITLE-ABS-KEY(diagnostic test) OR TITLE-ABS-KEY(Finnish Diabetes Risk Score) OR TITLE-ABS-KEY(FINDRISC) OR TITLE-ABS-KEY(Latin-American FINDRISC) OR TITLE-ABS-KEY(LA-FINDRISC)) AND TITLE-ABS-KEY("Antigua and Barbuda" OR "Argentina" OR "Bahamas" OR "Barbados" OR "Belize" OR "Bolivia" OR "Brazil" OR "United States Virgin Islands" OR "British Virgin Islands" OR "Chile" OR "Colombia" OR "Costa Rica" OR "Cuba" OR "Dominica" OR "Dominican Republic" OR "Ecuador" OR "El Salvador" OR "Grenada" OR "Guatemala" OR "Guyana" OR "Haiti" OR "Honduras" OR "Jamaica" OR "Mexico" OR "Nicaragua" OR "Panama" OR "Paraguay" OR "Peru" OR "Puerto Rico" OR "Saint Kitts and Nevis" OR "Saint Lucia" OR "Saint Vincent and the Grenadines" OR "Suriname" OR "Trinidad and Tobago" OR "West Indies" OR "Uruguay" OR "Venezuela" OR "Latin America" OR latin amer* OR "South America" OR south amer* OR "Central America" OR central amer* OR "Caribbean Region") AND NOT DBCOLL(medl)

# **Appendix S3** **Search terms used in LILACS**

((diabetes mellitus) OR (diabetes tipo 2) OR (diabetes mellitus tipo 2) OR (diabetes) OR (pre-diabetes) OR (pre-diabetic$) OR (estado prediabetico)) AND ((funcion de riesgo) OR (evaluacion del riesgo) OR (funcion$ de riesgo) OR (ecuacion de riesgo) OR (tabla de riesgos) OR (herramienta de ajuste de riesgo) OR (funcion de evaluación de riesgo) OR (asesor de riesgo) OR (calculo de riesgo) OR (calculadora de riesgo) OR (motor de riesgo) OR (umbral de riesgo) OR (metodo de calificacion de riesgo) OR (esquema de puntuacion) OR (sistema de puntuacion de riesgo) OR (prediccion de riesgo) OR (algoritmo de riesgo) OR (modelo predictivo) OR (prediccion de riesgo) OR (modelo de prediccion) OR (instrumento predictivo) OR (proyecto$ riesgo) OR (tamizaje) OR (escala de riesgo) OR (Finnish Diabetes Risk Score) OR (FINDRISC) OR (Latin-American FINDRISC) OR (LA-FINDRISC)) AND (("Antigua y Barbuda") OR ("Argentina") OR ("Aruba") OR ("Bahamas") OR ("Barbados") OR ("Belice") OR ("Bolivia") OR ("Brasil") OR ("Islas Vírgenes de los Estados Unidos") OR ("Islas Vírgenes Británicas") OR ("Islas Caimán") OR ("Chile") OR ("Colombia") OR ("Costa Rica") OR ("Cuba") OR ("Curazao") OR ("Dominica") OR ("Republica Dominicana") OR ("Ecuador") OR ("El Salvador") OR ("Granada") OR ("Guatemala") OR ("Guyana") OR ("Haití") OR ("Honduras") OR ("Jamaica") OR ("México") OR ("Nicaragua") OR ("Panamá") OR ("Paraguay") OR ("Perú") OR ("Puerto Rico") OR ("San Cristóbal y Nieves ") OR ("Santa Lucía") OR ("San Vicente y las Granadinas ") OR ("Surinam") OR ("Trinidad y Tobago") OR ("Turcas y Caicos ") OR ("Uruguay") OR ("Venezuela") OR ("América Latina") OR ("Latinoamérica") OR ("América del Sur") OR ("Sudamérica") OR ("Suramérica​") OR ("América Central") OR ("Centroamérica") OR ("América del Centro") OR ("Caribe"))

# **Appendix S4 Data extraction by domain**

Green shade signals the development models. NA=not applicable. NI=no information.

| Domains | | Source of data |
| --- | --- | --- |
|  | Study | Source of data |
| 1 | Pires de Sousa, 2009 - Derivation | Cross-sectional |
| 1 | Pires de Sousa, 2009 - Validation | Cross-sectional |
| 2 | Bernabe-Ortiz, 2016 - Derivation | Cross-sectional |
| 2 | Bernabe-Ortiz, 2016 - Validation (CS) | Case-control |
| 2 | Bernabe-Ortiz, 2016 - Validation(LON) | Cohort |
| 3 | Bernabe-Ortiz, 2018 - FINDRISC Simplified | Case-control |
| 3 | Bernabe-Ortiz, 2018 - FINDRISC | Cross-sectional |
| 3 | Bernabe-Ortiz, 2018 - LA-FINDRISC | Cross-sectional |
| 3 | Bernabe-Ortiz, 2018 - Peruvian Risk Score | Cross-sectional |
| 4 | Felix-Martinez, 2018 - Derivation 2006 | Cross-sectional |
| 4 | Felix-Martinez, 2018 - Derivation 2012 | Cross-sectional |
| 4 | Felix-Martinez, 2018 - Validation - 2006 | Cross-sectional |
| 4 | Felix-Martinez, 2018 - Validation - 2012 | Cross-sectional |
| 5 | Guerrero-Romero, 2010 - Derivation | Cross-sectional |
| 5 | Guerrero-Romero, 2010 - Validation | Cohort |

| Domains | | Participants | | | | | | | | |
| --- | --- | --- | --- | --- | --- | --- | --- | --- | --- | --- |
|  | Study | Participant location | Baseline year | End year (cohorts) | Sampling | Inclusion criteria | Exclusion criteria | Outcome prevalence (%) | Baseline mean age | Baseline % men |
| 1 | Pires de Sousa, 2009 - Derivation | Community |  |  | Random | General population aged 25-64 years | None reported | 9.60 | 48.88 | 45.22 |
| 1 | Pires de Sousa, 2009 - Validation | Community |  |  | Random | People aged ≥15 years old whose birthday was close to the interview date | None reported | 6.21 | 52.37 | 25.73 |
| 2 | Bernabe-Ortiz, 2016 - Derivation | Community | 2004-05 |  | Random | People aged ≥20 years old, habitual residents and able to provide informed consent | Pregnant women and currently breastfeeding | 2.00 | 50.50 | 51.10 |
| 2 | Bernabe-Ortiz, 2016 - Validation (CS) | Community | 2010 |  | Random | People aged ≥35 years old, habitual residents and able to provide informed consent | None reported | 2.90 | 55.30 | 49.10 |
| 2 | Bernabe-Ortiz, 2016 - Validation(LON) | Community | 2010 | 2014 | Random | People aged ≥35 years old, habitual residents and able to provide informed consent | None reported | 2.90 | 55.30 | 49.10 |
| 3 | Bernabe-Ortiz, 2018 - FINDRISC Simplified | Community |  |  | Random | People aged 30-69 years, full time residents and able to understand procedures and provide informed consent | Pregnant women and people with any physical disability preventing clinical (e.g., anthropometrics) assessment | 4.41 | 48.20 | 49.70 |
| 3 | Bernabe-Ortiz, 2018 - FINDRISC | Community |  |  | Random | People aged 30-69 years, full time residents and able to understand procedures and provide informed consent | Pregnant women and people with any physical disability preventing clinical (e.g., anthropometrics) assessment | 4.41 | 48.20 | 49.70 |
| 3 | Bernabe-Ortiz, 2018 - LA-FINDRISC | Community |  |  | Random | People aged 30-69 years, full time residents and able to understand procedures and provide informed consent | Pregnant women and people with any physical disability preventing clinical (e.g., anthropometrics) assessment | 4.41 | 48.20 | 49.70 |
| 3 | Bernabe-Ortiz, 2018 - Peruvian Risk Score | Community |  |  | Random | People aged 30-69 years, full time residents and able to understand procedures and provide informed consent | Pregnant women and people with any physical disability preventing clinical (e.g., anthropometrics) assessment | 4.41 | 48.20 | 49.70 |
| 4 | Felix-Martinez, 2018 - Derivation 2006 | Community | 2006 |  | Random |  |  | 3.00 | 42.14 | 38.00 |
| 4 | Felix-Martinez, 2018 - Derivation 2012 | Community | 2012 |  | Random |  |  | 1.25 | 43.08 | 38.31 |
| 4 | Felix-Martinez, 2018 - Validation - 2006 | Community | 2000 |  | Random |  |  | 2.01 | 40.84 | 29.40 |
| 4 | Felix-Martinez, 2018 - Validation - 2012 | Community | 2000 |  | Random |  |  | 2.01 | 40.84 | 29.40 |
| 5 | Guerrero-Romero, 2010 - Derivation | Community |  |  |  |  |  |  |  |  |
| 5 | Guerrero-Romero, 2010 - Validation | Community | 1996 |  | Random | Healthy adults aged 20-65 | People with history of diabetes or those cases detected at the baseline assessment | 14.16 |  | 29.90 |

| Domains | | Outcome | | | | | |
| --- | --- | --- | --- | --- | --- | --- | --- |
|  | Study | Outcome | Outcome details | Same outcome definition for all patients? | Blinded outcome | Predictors part of the outcome | Mean follow-up (years) |
| 1 | Pires de Sousa, 2009 - Derivation | Diabetes lab-only | Fasting plasma glucose ≥126 mg/dL (7.0 mmol/L) | Yes | Yes | No | NA |
| 1 | Pires de Sousa, 2009 - Validation | Diabetes lab-only | Fasting plasma glucose ≥126 mg/dL (7.0 mmol/L) | Yes | Yes | No | NA |
| 2 | Bernabe-Ortiz, 2016 - Derivation | Diabetes lab-only | Fasting plasma glucose ≥7.0 mmol/L (126 mg/dL) | Yes | Yes | Yes | NA |
| 2 | Bernabe-Ortiz, 2016 - Validation (CS) | Diabetes lab-only | Fasting plasma glucose ≥7.0 mmol/L (126 mg/dL) | Yes | Yes | Yes | NA |
| 2 | Bernabe-Ortiz, 2016 - Validation(LON) | Diabetes lab-only | Fasting plasma glucose ≥7.0 mmol/L (126 mg/dL) | Yes | Yes | Yes | 2.5 |
| 3 | Bernabe-Ortiz, 2018 - FINDRISC Simplified | Diabetes lab-only | Fasting glucose ≥126 mg/dL (≥7.0 mmol/L) or 2-hour plasma glucose ≥200 mg/dL (≥11.1 mmol/L) | Yes | Yes | Yes | NA |
| 3 | Bernabe-Ortiz, 2018 - FINDRISC | Diabetes lab-only | Fasting glucose ≥126 mg/dL (≥7.0 mmol/L) or 2-hour plasma glucose ≥200 mg/dL (≥11.1 mmol/L) | Yes | Yes | No | NA |
| 3 | Bernabe-Ortiz, 2018 - LA-FINDRISC | Diabetes lab-only | Fasting glucose ≥126 mg/dL (≥7.0 mmol/L) or 2-hour plasma glucose ≥200 mg/dL (≥11.1 mmol/L) | Yes | Yes | No | NA |
| 3 | Bernabe-Ortiz, 2018 - Peruvian Risk Score | Diabetes lab-only | Fasting glucose ≥126 mg/dL (≥7.0 mmol/L) or 2-hour plasma glucose ≥200 mg/dL (≥11.1 mmol/L) | Yes | Yes | No | NA |
| 4 | Felix-Martinez, 2018 - Derivation 2006 | Diabetes lab-only | Fasting plasma glucose ≥126 mg/dL or a random glucose ≥200 mg/dL | Yes | Yes | No | NA |
| 4 | Felix-Martinez, 2018 - Derivation 2012 | Diabetes lab-only | Fasting plasma glucose ≥126 mg/dL or a random glucose ≥200 mg/dL | Yes | Yes | No | NA |
| 4 | Felix-Martinez, 2018 - Validation - 2006 | Diabetes lab-only | Fasting plasma glucose ≥126 mg/dL or a random glucose ≥200 mg/dL | Yes | Yes | No | NA |
| 4 | Felix-Martinez, 2018 - Validation - 2012 | Diabetes lab-only | Fasting plasma glucose ≥126 mg/dL or a random glucose ≥200 mg/dL | Yes | Yes | No | NA |
| 5 | Guerrero-Romero, 2010 - Derivation |  | New diagnosis of diabetes according to American Diabetes Association criteria | Yes | Yes | Yes | NA |
| 5 | Guerrero-Romero, 2010 - Validation |  | New diagnosis of diabetes according to American Diabetes Association criteria | Yes | Yes | Yes | 7.0 |

| Domains | | Candidate Predictors | | | | | | |
| --- | --- | --- | --- | --- | --- | --- | --- | --- |
|  | Study | Number of candidate predictors | Number of predictors in the final model | Predictors timing | List of predictors in the final model | Predictors definition | Predictors ascertainment | Predictors modelling |
| 1 | Pires de Sousa, 2009 - Derivation | 19 | 5 | Baseline | Age, BMI, Hypertension | Age [35-44 (ref), 45-54, ≥55]; BMI [<25 (ref), 25-29.9, ≥30]; Hypertension [no (ref), yes] | Participants were invited for clinical examination. Hypertension was >140/90 mmHg or use of antihypertensive drugs | Continuous variables were categorized into bands |
| 1 | Pires de Sousa, 2009 - Validation |  | 5 | Baseline | Age, BMI, Hypertension | Age [35-44 (ref), 45-54, ≥55]; BMI [<25 (ref), 25-29.9, ≥30]; Hypertension [no (ref), yes] | Participants were invited for clinical examination. Hypertension was >140/90 mmHg or use of antihypertensive drugs | Continuous variables were categorized into bands |
| 2 | Bernabe-Ortiz, 2016 - Derivation | 14 | 4 | Baseline | Age; diabetes in first-degree relatives; waist circumference | Age [<55 (ref), ≥55]; diabetes in relatives [versus no diabetes in relatives]; waist circumference [<90 (ref), 90.0-99.9, ≥100] | Questionnaires and clinical evaluation (anthropometrics) | Continuous variables were categorized |
| 2 | Bernabe-Ortiz, 2016 - Validation (CS) |  | 4 | Baseline | Age; diabetes in first-degree relatives; waist circumference | Age [<55 (ref), ≥55]; diabetes in relatives [versus no diabetes in relatives]; waist circumference [<90 (ref), 90.0-99.9, ≥100] | Questionnaires and clinical evaluation (anthropometrics) | Continuous variables were categorized |
| 2 | Bernabe-Ortiz, 2016 - Validation(LON) |  | 4 | Baseline | Age; diabetes in first-degree relatives; waist circumference | Age [<55 (ref), ≥55]; diabetes in relatives [versus no diabetes in relatives]; waist circumference [<90 (ref), 90.0-99.9, ≥100] | Questionnaires and clinical evaluation (anthropometrics) | Continuous variables were categorized |
| 3 | Bernabe-Ortiz, 2018 - FINDRISC Simplified | 12 | 5 | Baseline | Waist circumference; blood pressure medication; history of high blood glucose levels; family history of type 2 diabetes mellitus | Waist circumference [women <80 & men <94 (ref), women 80-87, & men 94-101, women ≥88 & men ≥102]; blood pressure medication [no (ref), yes]; history of high blood glucose levels [no (ref), yes]; family history of type 2 diabetes mellitus [no (ref), parent/siblings/offspring] | Questionnaires and clinical evaluation (anthropometrics) | Continuous variables were categorized |
| 3 | Bernabe-Ortiz, 2018 - FINDRISC |  | 13 | Baseline | Age; body mass index; waist circumference; physical activity; fruits and vegetable intake; regular medication for hypertension; history of high glucose levels; diabetes in relatives | Age [<45 (ref), 45-54, 55-64, ≥65]; body mass index [<25 (ref), 25.-29.99, ≥30]; waist circumference [men <94 & women <80, men 94-102 & women 80-88, men >102 & women >88]; physical activity at least 30 min/day [yes (ref), no]; Fruits and vegetables intake [every day (ref), not every day]; regular medication for hypertension [no (ref), yes]; history of high glucose levels [no (ref), yes]; diabetes in relatives [no, (ref), yes in grandparents/cousins/uncle/aunt, yes in parents/siblings/offspring] | Questionnaires and clinical evaluation (anthropometrics) |  |
| 3 | Bernabe-Ortiz, 2018 - LA-FINDRISC |  | 12 | Baseline | Age; body mass index; waist circumference; physical activity; fruits and vegetable intake; regular medication for hypertension; history of high glucose levels; diabetes in relatives | Age [<45 (ref), 45-54, 55-64, ≥65]; body mass index [<25 (ref), 25.-29.99, ≥30]; waist circumference [men <94 & women <80, men 94-102 & women 80-88]; physical activity at least 30 min/day [yes (ref), no]; Fruits and vegetables intake [every day (ref), not every day]; regular medication for hypertension [no (ref), yes]; history of high glucose levels [no (ref), yes]; diabetes in relatives [no, (ref), yes in grandparents/cousins/uncle/aunt, yes in parents/siblings/offspring] | Questionnaires and clinical evaluation (anthropometrics) |  |
| 3 | Bernabe-Ortiz, 2018 - Peruvian Risk Score |  | 4 | Baseline | Age; diabetes in first-degree relatives; waist circumference | Age [<55 (ref), ≥55]; diabetes in relatives [versus no diabetes in relatives]; waist circumference [<90 (ref), 90.0-99.9, ≥100] | Questionnaires and clinical evaluation (anthropometrics) |  |
| 4 | Felix-Martinez, 2018 - Derivation 2006 | 13 | 3 | Baseline | Age; systolic blood pressure; waist circumference |  | Questionnaires and clinical evaluation (anthropometrics) | Numeric variables were treated as such |
| 4 | Felix-Martinez, 2018 - Derivation 2012 | 13 | 4 | Baseline | Age, family history of diabetes, height, waist circumference |  | Questionnaires and clinical evaluation (anthropometrics) | Only family history of diabetes seems to be a dichotomic variable, numeric variables were treated as such |
| 4 | Felix-Martinez, 2018 - Validation - 2006 |  | 3 | Baseline | Age; systolic blood pressure; waist circumference |  | Questionnaires and clinical evaluation (anthropometrics) | Numeric variables were treated as such |
| 4 | Felix-Martinez, 2018 - Validation - 2012 |  | 4 | Baseline | Age, family history of diabetes, height, waist circumference |  | Questionnaires and clinical evaluation (anthropometrics) | Only family history of diabetes seems to be a dichotomic variable, numeric variables were treated as such |
| 5 | Guerrero-Romero, 2010 - Derivation | 11 | 11 | Baseline | Sex; age; family history of diabetes; family history of hypertension; family history of obesity; personal history of gestational diabetes or macrosomia; sedentarism; hypertension; body mass index (obesity); fasting glucose; triglycerides | Family history refers to a first-degree relative; Hypertension [systolic ≥135 or diastolic ≥85]; sedentarism was the lack of planned physical activity or when physical activity was <50 min/week; gestational diabetes if during the previous pregnancy she had two fasting glucose ≥7 mmol/L (126 mg/dL) after week 24, one fasting glucose ≥11.1 mmol/L (200 mg/dL) o a glucose measure of ≥ 8.6 mmol/L (155 mg/dL) after a 50 gram glucose load; macrosomia was present if she had a previous delivery with weight ≥4 kg; overweight as body mass index ≥25 and obesity as body mass index ≥30; high glucose values if a fasting glucose was ≥5.6 mmol/L (100 mg/dL); hypertriglyceridemia when these were ≥1.7 mmol/L (150 mg/dL) | NI | Continuous variables were categorized |
| 5 | Guerrero-Romero, 2010 - Validation |  | 11 | Baseline | Sex; age; family history of diabetes; family history of hypertension; family history of obesity; personal history of gestational diabetes or macrosomia; sedentarism; hypertension; body mass index (obesity); fasting glucose; triglycerides | Family history refers to a first-degree relative; Hypertension [systolic ≥135 or diastolic ≥85]; sedentarism was the lack of planned physical activity or when physical activity was <50 min/week; gestational diabetes if during the previous pregnancy she had two fasting glucose ≥7 mmol/L (126 mg/dL) after week 24, one fasting glucose ≥11.1 mmol/L (200 mg/dL) o a glucose measure of ≥ 8.6 mmol/L (155 mg/dL) after a 50 gram glucose load; macrosomia was present if she had a previous delivery with weight ≥4 kg; overweight as body mass index ≥25 and obesity as body mass index ≥30; high glucose values if a fasting glucose was ≥5.6 mmol/L (100 mg/dL); hypertriglyceridemia when these were ≥1.7 mmol/L (150 mg/dL) | NI | Continuous variables were categorized |

| Domains | | Sample Size | | | Missing Data | | |
| --- | --- | --- | --- | --- | --- | --- | --- |
|  | Study | Baseline sample size | Number of outcome events | Total outcome events per candidate predictors | Missing data | Number of participants with missing data | Missing data per predictors |
| 1 | Pires de Sousa, 2009 - Derivation | 1224 | 118 | 6.21 | Complete-case | 8 | 1.60 |
| 1 | Pires de Sousa, 2009 - Validation | 467 | 29 |  | Complete-case | 91 | 18.20 |
| 2 | Bernabe-Ortiz, 2016 - Derivation | 2472 | 48 | 3.43 | Complete-case | 129 | 32.25 |
| 2 | Bernabe-Ortiz, 2016 - Validation (CS) | 2948 | 85 |  | Complete-case | 465 | 116.25 |
| 2 | Bernabe-Ortiz, 2016 - Validation(LON) | 2577 | 121 |  | Complete-case |  | 0.00 |
| 3 | Bernabe-Ortiz, 2018 - FINDRISC Simplified | 1609 | 71 | 5.92 | Complete-case | 3 | 0.60 |
| 3 | Bernabe-Ortiz, 2018 - FINDRISC | 1609 | 71 |  | Complete-case | 3 | 0.23 |
| 3 | Bernabe-Ortiz, 2018 - LA-FINDRISC | 1609 | 71 |  | Complete-case | 3 | 0.25 |
| 3 | Bernabe-Ortiz, 2018 - Peruvian Risk Score | 1609 | 71 |  | Complete-case | 3 | 0.75 |
| 4 | Felix-Martinez, 2018 - Derivation 2006 | 6995 | 207 | 15.92 | Complete-case |  | 0.00 |
| 4 | Felix-Martinez, 2018 - Derivation 2012 | 4083 | 51 | 3.92 | Complete-case |  | 0.00 |
| 4 | Felix-Martinez, 2018 - Validation - 2006 | 28913 | 582 |  | Complete-case |  | 0.00 |
| 4 | Felix-Martinez, 2018 - Validation - 2012 | 28913 | 582 |  | Complete-case |  | 0.00 |
| 5 | Guerrero-Romero, 2010 - Derivation | 711 |  | 0 | NI |  | 0.00 |
| 5 | Guerrero-Romero, 2010 - Validation | 438 | 62 |  | Complete-case | 87 | 7.91 |

| Domains | | Model Development | | | | | |
| --- | --- | --- | --- | --- | --- | --- | --- |
|  | Study | Regression method | Were the model assumptions verified? | Predictors selection | If the prediction model was a replication, which was the original model? | If there were pre-selection, describe the method | Was a shrinkage method used? |
| 1 | Pires de Sousa, 2009 - Derivation | Logistic | NI | Pre-selection |  | Stepwise backward elimination | No |
| 1 | Pires de Sousa, 2009 - Validation | Logistic | NI | Pre-selection |  | Stepwise backward elimination | No |
| 2 | Bernabe-Ortiz, 2016 - Derivation | Logistic | NI | Pre-selection |  | Stepwise backward elimination | No |
| 2 | Bernabe-Ortiz, 2016 - Validation (CS) |  |  |  |  |  |  |
| 2 | Bernabe-Ortiz, 2016 - Validation(LON) |  |  |  |  |  |  |
| 3 | Bernabe-Ortiz, 2018 - FINDRISC Simplified | Logistic | NI | Pre-selection |  | Stepwise backward elimination | No |
| 3 | Bernabe-Ortiz, 2018 - FINDRISC |  |  |  |  |  | No |
| 3 | Bernabe-Ortiz, 2018 - LA-FINDRISC |  |  |  |  |  | No |
| 3 | Bernabe-Ortiz, 2018 - Peruvian Risk Score |  |  |  |  |  | No |
| 4 | Felix-Martinez, 2018 - Derivation 2006 | Logistic | NI | Pre-selection |  | Stepwise backward elimination | No |
| 4 | Felix-Martinez, 2018 - Derivation 2012 | Logistic | NI | Pre-selection |  | Stepwise backward elimination | No |
| 4 | Felix-Martinez, 2018 - Validation - 2006 |  |  |  |  |  |  |
| 4 | Felix-Martinez, 2018 - Validation - 2012 |  |  |  |  |  |  |
| 5 | Guerrero-Romero, 2010 - Derivation | Logistic | NI | All |  |  | No |
| 5 | Guerrero-Romero, 2010 - Validation |  |  |  |  |  |  |

| Domains | | Model Performance | | | | |
| --- | --- | --- | --- | --- | --- | --- |
|  | Study | Calibration | Discrimination | Classification measures | Cut-off point | For replication studies, was the cut-off the same? |
| 1 | Pires de Sousa, 2009 - Derivation | NI | 0.772 | Classification measures are presented for several thresholds. E.g., for a threshold = 18, sensitivity=0.7586; specificity=0.6685; positive predictive value=0.197; negative predictive value=0.963; accuracy=0.68; needing to additional test=0.372 | 18 |  |
| 1 | Pires de Sousa, 2009 - Validation | NI | 0.720 | Classification measures are presented for several thresholds. E.g., for a threshold = 18, sensitivity=0.8571; specificity=0.4479; positive predictive value=0.093; negative predictive value=0.979; accuracy=0.47; needing to additional test=0.571 | 18 |  |
| 2 | Bernabe-Ortiz, 2016 - Derivation | Hosmer-Lemeshow test, p=0.21 | 0.720 | Classification measures are presented for several thresholds. E.g., for a threshold = 2, sensitivity=0.696; specificity=0.658; positive predictive value=0.039; negative predictive value=0.991 | 2 |  |
| 2 | Bernabe-Ortiz, 2016 - Validation (CS) |  | 0.680 | Classification measures are presented for several thresholds. E.g., for a threshold = 2, sensitivity=0.702; specificity=0.589; positive predictive value=0.048; negative predictive value=0.985 | 2 |  |
| 2 | Bernabe-Ortiz, 2016 - Validation(LON) |  | 0.660 | Classification measures are presented for several thresholds. E.g., for a threshold = 2, sensitivity=0.694; specificity=0.589; positive predictive value=0.078; negative predictive value=0.974 | 2 |  |
| 3 | Bernabe-Ortiz, 2018 - FINDRISC Simplified |  | 0.710 | Sensitivity=0.859; specificity=0.467; positive predictive value=0.074; negative predictive value=0.985; likelihood ratio positive=1.6; likelihood ratio negative=0.3; diagnostic odd ratio=5.3 | 3 |  |
| 3 | Bernabe-Ortiz, 2018 - FINDRISC |  | 0.690 | Sensitivity=0.690; specificity=0.668; positive predictive value=0.094; negative predictive value=0.978; likelihood ratio positive=2.1; likelihood ratio negative=0.5; diagnostic odd ratio=4.5 | 11 | No |
| 3 | Bernabe-Ortiz, 2018 - LA-FINDRISC |  | 0.680 | Sensitivity=0.704; specificity=0.591; positive predictive value=0.079; negative predictive value=0.970; likelihood ratio positive=1.7; likelihood ratio negative=0.5; diagnostic odd ratio=3.4 | 10 | No |
| 3 | Bernabe-Ortiz, 2018 - Peruvian Risk Score |  | 0.640 | Sensitivity=0.648; specificity=0.537; positive predictive value=0.064; negative predictive value=0.968; likelihood ratio positive=1.4; likelihood ratio negative=0.7; diagnostic odd ratio=2.1 | 2 | Yes |
| 4 | Felix-Martinez, 2018 - Derivation 2006 |  | 0.700 | Sensitivity=0.750; specificity=0.610; cut-off probability value=0.027 | 0.027 |  |
| 4 | Felix-Martinez, 2018 - Derivation 2012 |  | 0.660 | Sensitivity=0.750; specificity=0.560; cut-off probability value=0.010 | 0.010 |  |
| 4 | Felix-Martinez, 2018 - Validation - 2006 |  | 0.730 | Sensitivity=0.740; specificity=0.620 |  |  |
| 4 | Felix-Martinez, 2018 - Validation - 2012 |  | 0.710 | Sensitivity=0.760; specificity=0.550 |  |  |
| 5 | Guerrero-Romero, 2010 - Derivation |  |  |  |  |  |
| 5 | Guerrero-Romero, 2010 - Validation |  | 0.910 | Sensitivity=0.920; specificity=0.710; positive predictive value=0.350; negative predictive value=0.975 |  |  |

| Domains | | Model Evaluation | | | Results | | | | Discussion | | |
| --- | --- | --- | --- | --- | --- | --- | --- | --- | --- | --- | --- |
|  | Study | Internal validation | External validation | If applicable, details of external validation | Was a simplified model presented? | Were the coefficients of the regression model presented? | Was the baseline risk presented? | Were there alternative results presentation? | Interpretation of the results | Comparison with other studies in LAC | Generalizability |
| 1 | Pires de Sousa, 2009 - Derivation | NI | Yes | External validation in an independent population-based database | Yes | Yes | No | Yes | Confirmatory | No | NI |
| 1 | Pires de Sousa, 2009 - Validation |  |  |  |  |  |  |  |  |  |  |
| 2 | Bernabe-Ortiz, 2016 - Derivation | Bootstrap | Yes | External validation in an independent population-based database | Yes | Yes | No | Yes | Exploratory | Yes | Non-generalizability |
| 2 | Bernabe-Ortiz, 2016 - Validation (CS) |  |  |  |  |  |  |  |  |  |  |
| 2 | Bernabe-Ortiz, 2016 - Validation(LON) |  |  |  |  |  |  |  |  |  |  |
| 3 | Bernabe-Ortiz, 2018 - FINDRISC Simplified | NI | No |  | Yes | Yes | No | Yes | Exploratory | Yes | Non-generalizability |
| 3 | Bernabe-Ortiz, 2018 - FINDRISC |  |  |  |  |  |  |  |  |  |  |
| 3 | Bernabe-Ortiz, 2018 - LA-FINDRISC |  |  |  |  |  |  |  |  |  |  |
| 3 | Bernabe-Ortiz, 2018 - Peruvian Risk Score |  |  |  |  |  |  |  |  |  |  |
| 4 | Felix-Martinez, 2018 - Derivation 2006 | Cross-validation | Yes | External validation in an independent population-based database | Yes | Yes | Yes | Yes | NI | Yes | NI |
| 4 | Felix-Martinez, 2018 - Derivation 2012 | Cross-validation | Yes | External validation in an independent population-based database | Yes | Yes | Yes | Yes | NI | Yes | NI |
| 4 | Felix-Martinez, 2018 - Validation - 2006 |  |  |  |  |  |  |  |  |  |  |
| 4 | Felix-Martinez, 2018 - Validation - 2012 |  |  |  |  |  |  |  |  |  |  |
| 5 | Guerrero-Romero, 2010 - Derivation | Cross-validation | Yes | External validation in an independent population-based database | No | No | No | Yes | Confirmatory | No | Non-generalizability |
| 5 | Guerrero-Romero, 2010 - Validation |  |  |  |  |  |  |  |  |  |  |

# **Appendix S5 Risk of bias assessment (PROBAST)**

Y=yes; N=no; n/a=not applicable; NI=no information

| **Study** | **Participants** | | **Predictors** | | |
| --- | --- | --- | --- | --- | --- |
|  | **Were appropriate data sources used, e.g., cohort, RCT, or nested case–control study data?** | **Were all inclusions and exclusions of participants appropriate?** | **Were predictors defined and assessed in a similar way for all participants?** | **Were predictor assessments made without knowledge of outcome data?** | **Are all predictors available at the time the model is intended to be used?** |
| Bernabe-Ortiz, 2018 (FINDRISC) | Y | Y | Y | Y | Y |
| Bernabe-Ortiz, 2018 (LA-FINDRISC) | Y | Y | Y | Y | Y |
| Bernabe-Ortiz, 2018 (Peruvian) | Y | Y | Y | Y | Y |
| Bernabe-Ortiz, 2018 (Simplified FINDRISC) | Y | Y | Y | Y | Y |
| Felix-Martinez, 2018 (Derivation NHNS-2006) | Y | Y | Y | NI | Y |
| Felix-Martinez, 2018 (Derivation NHNS-2012) | Y | Y | Y | NI | Y |
| Felix-Martinez, 2018 (Validation NHNS-2006) | Y | Y | Y | NI | Y |
| Felix-Martinez, 2018 (Validation NHNS-2012) | Y | Y | Y | NI | Y |
| Guerrero-Romero, 2010 (Derivation) | Y | NI | PY | NI | N |
| Guerrero-Romero, 2010 (Validation) | Y | Y | PY | NI | N |
| Pires de Sousa, 2009 - Derivation | PY | Y | Y | PY | Y |
| Pires de Sousa, 2009 - Validation | PY | Y | Y | PY | Y |
| Bernabe-Ortiz, 2016 - Derivation | PY | Y | Y | Y | Y |
| Bernabe-Ortiz, 2016 - Validation | PY | Y | Y | Y | Y |

| **Study** | **Outcome** | | | | | |
| --- | --- | --- | --- | --- | --- | --- |
|  | **Was the outcome determined appropriately?** | **Was a prespecified or standard outcome definition used?** | **Were predictors excluded from the outcome definition?** | **Was the outcome defined and determined in a similar way for all participants?** | **Was the outcome determined without knowledge of predictor information?** | **Was the time interval between predictor assessment and outcome determination appropriate?** |
| Bernabe-Ortiz, 2018 (FINDRISC) | Y | Y | Y | Y | Y | Y |
| Bernabe-Ortiz, 2018 (LA-FINDRISC) | Y | Y | Y | Y | Y | Y |
| Bernabe-Ortiz, 2018 (Peruvian) | Y | Y | Y | Y | Y | Y |
| Bernabe-Ortiz, 2018 (Simplified FINDRISC) | Y | Y | Y | Y | Y | Y |
| Felix-Martinez, 2018 (Derivation NHNS-2006) | Y | Y | Y | Y | NI | PY |
| Felix-Martinez, 2018 (Derivation NHNS-2012) | Y | Y | Y | Y | NI | PY |
| Felix-Martinez, 2018 (Validation NHNS-2006) | Y | Y | Y | Y | NI | PY |
| Felix-Martinez, 2018 (Validation NHNS-2012) | Y | Y | Y | Y | NI | PY |
| Guerrero-Romero, 2010 (Derivation) | PY | PY | N | PY | NI | Y |
| Guerrero-Romero, 2010 (Validation) | PY | PY | N | PY | NI | Y |
| Pires de Sousa, 2009 - Derivation | Y | Y | Y | Y | Y | PY |
| Pires de Sousa, 2009 - Validation | Y | Y | Y | Y | Y | PY |
| Bernabe-Ortiz, 2016 - Derivation | Y | Y | PN | Y | Y | PY |
| Bernabe-Ortiz, 2016 - Validation | Y | Y | PN | Y | Y | PY |

| **Study** | **Analysis** | | | | |
| --- | --- | --- | --- | --- | --- |
|  | **Were there a reasonable number of participants with the outcome?** | **Were continuous and categorical predictors handled appropriately?** | **Were all enrolled participants included in the analysis?** | **Were participants with missing data handled appropriately?** | **Was selection of predictors based on univariable analysis avoided? [development studies only]** |
| Bernabe-Ortiz, 2018 (FINDRISC) | N | Y | Y | PY | n/a |
| Bernabe-Ortiz, 2018 (LA-FINDRISC) | N | Y | Y | PY | n/a |
| Bernabe-Ortiz, 2018 (Peruvian) | N | Y | Y | PY | n/a |
| Bernabe-Ortiz, 2018 (Simplified FINDRISC) | N | Y | Y | PY | Y |
| Felix-Martinez, 2018 (Derivation NHNS-2006) | N | Y | Y | N | Y |
| Felix-Martinez, 2018 (Derivation NHNS-2012) | N | Y | Y | N | Y |
| Felix-Martinez, 2018 (Validation NHNS-2006) | Y | Y | Y | N | n/a |
| Felix-Martinez, 2018 (Validation NHNS-2012) | Y | Y | Y | N | n/a |
| Guerrero-Romero, 2010 (Derivation) | NI | Y | NI | NI | Y |
| Guerrero-Romero, 2010 (Validation) | N | Y | PN | NI | n/a |
| Pires de Sousa, 2009 - Derivation | Y | Y | NI | PN | Y |
| Pires de Sousa, 2009 - Validation | PN | Y | NI | PN | n/a |
| Bernabe-Ortiz, 2016 - Derivation | PY | Y | NI | PN | Y |
| Bernabe-Ortiz, 2016 - Validation | PN | Y | NI | PN | n/a |

| **Study** | **Analysis** | | | |
| --- | --- | --- | --- | --- |
|  | **Were complexities in the data (e.g., censoring, competing risks, sampling of control participants) accounted for appropriately?** | **Were relevant model performance measures evaluated appropriately?** | **Were model overfitting and optimism in model performance accounted for? [development studies only]** | **Do predictors and their assigned weights in the final model correspond to the results from the reported multivariable analysis? [development studies only]** |
| Bernabe-Ortiz, 2018 (FINDRISC) | Y | N | n/a | n/a |
| Bernabe-Ortiz, 2018 (LA-FINDRISC) | Y | N | n/a | n/a |
| Bernabe-Ortiz, 2018 (Peruvian) | Y | N | n/a | n/a |
| Bernabe-Ortiz, 2018 (Simplified FINDRISC) | Y | N | PY | Y |
| Felix-Martinez, 2018 (Derivation NHNS-2006) | Y | N | Y | Y |
| Felix-Martinez, 2018 (Derivation NHNS-2012) | Y | N | Y | Y |
| Felix-Martinez, 2018 (Validation NHNS-2006) | Y | N | n/a | n/a |
| Felix-Martinez, 2018 (Validation NHNS-2012) | Y | N | n/a | n/a |
| Guerrero-Romero, 2010 (Derivation) | NI | N | NI | NI |
| Guerrero-Romero, 2010 (Validation) | N | N | n/a | Y |
| Pires de Sousa, 2009 - Derivation | PY | PN | NI | Y |
| Pires de Sousa, 2009 - Validation | PY | PN | n/a | n/a |
| Bernabe-Ortiz, 2016 - Derivation | PY | PY | Y | Y |
| Bernabe-Ortiz, 2016 - Validation | Y | PY | n/a | n/a |
